# Systemic hypertension associated retinal microvascular changes can be detected with optical coherence tomography angiography

**DOI:** 10.1038/s41598-020-66736-w

**Published:** 2020-06-12

**Authors:** Christopher Sun, Carlo Ladores, Jimmy Hong, Duc Quang Nguyen, Jacqueline Chua, Daniel Ting, Leopold Schmetterer, Tien Yin Wong, Ching-Yu Cheng, Anna C. S. Tan

**Affiliations:** 1 Singapore National Eye Centre, Singapore, Singapore; 2 University of Santo Tomas Hospital, Manila, Philippines; 3 Singapore Eye Research Institute, Singapore, Singapore

**Keywords:** Hypertension, Eye manifestations, Medical research

## Abstract

A major complication of hypertension is microvascular damage and capillary rarefaction is a known complication of hypertensive end-organ damage which confers a higher risk of systemic disease such as stroke and cardiovascular events. Our aim was to study the effect of hypertension on the retinal microvasculature using non-invasive optical coherence tomography angiography (OCTA). We performed a case-control study of 94 eyes of 94 participants with systemic hypertension and 46 normal control eyes from the Singapore Chinese Eye Study using a standardized protocol to collect data on past medical history of hypertension, including the number and type of hypertensive medications and assessed mean arterial pressure. Retinal vascular parameters were measured in all eyes using OCTA. In the multivariate analysis adjusting for confounders, compared to controls, eyes of hypertensive patients showed a decrease in the macular vessel density at the level of the superficial [OR 0.02; 95% CI, 0 to 0.64; P 0.027] and deep venous plexuses [OR 0.03; 95% CI, 0 to 0.41; P 0.009] and an increase in the deep foveal avascular zone. This shows that hypertension is associated with reduced retinal vessel density and an increased foveal avascular zone, especially in the deep venous plexus, as seen on OCTA and there is a potential role in using OCTA as a clinical tool to monitor hypertensive damage and identifying at risk patients

## Introduction

Hypertension is a major cause of morbidity and mortality globally^1,2^, affecting 29.2% men and 24.8% women in 2012^3^. A major complication of hypertension is microvascular damage, related partly to abnormal vasomotor tone and increased wall-to-lumen ratio in relation to higher blood pressure^4,5^. It has also been suggested that vascular rarefaction may due to either functional alterations such as microvessel constriction resulting in non-perfusion or anatomical alterations resulting in actual non-perfusion and vessel loss^4^.

There have been many previous studies on the association between hypertension and retinal vasculature. Retinal fundus photo changes seen in response to hypertension include classic hypertensive retinopathy signs such as arteriovenous (AV) nicking, generalized or focal arteriolar narrowing, microaneurysms, intraretinal hemorrhages, cotton wool spots and papilloedema^6^. These changes have been shown to confer a higher risk of systemic disease such as stroke and cardiovascular events^6–9^. Other studies have also looked at larger retinal vessels (200–300 µm) using color fundus photos and demonstrated a correlation between narrower arteriolar diameter in hypertension^10–13^.

The impact of hypertension on capillary microvasculature is less clear. Historically, invasive fundus fluorescein angiography (FFA) was needed to evaluate perfusion and the structure of capillary microvasculature. Previous studies using FFA have shown that in patients with hypertension, there is an increase in perifoveal inter-capillary area and decrease in capillary blood velocity^14^. However, FFA is not suitable for use in large cohorts because it is invasive, requiring intravenous access, and has well-known side effects like nausea, vomiting and in rare instances anaphylactic reactions.

Optical coherence tomography angiography (OCTA) is a new, quick, non-invasive imaging modality that allows both the microvasculature within the retinal and choroidal layers to be studied in large populations of patients^15–17^. OCTA has the advantage over conventional FFA as it can image retinal vasculature in 3-dimensions with higher resolution, delineate the foveal avascular zone (FAZ) more accurately and can produce depth resolved images of the superficial and deep vascular plexi^15,17^. There are a few OCTA studies that have been published about retinal microvasculature changes related to systemic hypertension. Lee *et al*. examined only the superficial capillary plexus and showed that in patients with chronic hypertension and hypertensive retinopathy, there was a reduction in the foveal vessel density, perfusion density, mean ganglion cell-inner plexiform layer(GC-IPL) thickness, central foveal thickness (CFT) and retinal nerve fibre layer thickness (RNFL) and an increase in the FAZ, compared to their normotensive control group. Another study by Chua *et al*. examining the superficial vascular plexus (SVP) and deep vascular plexus (DVP) showed that in patients with poorly controlled blood pressure (BP) ≥ 140/90 mmHg, higher systolic BP and mean arterial pressure and a lower estimated glomerular filtration rate had a reduced DVP capillary density^18^.

The aim of our current study was to measure retinal vascular density and perfusion using OCTA in individuals with chronic treated hypertension. We hypothesize that OCTA will show subclinical alterations in retinal microvasculature parameters in both the superficial and deep plexi when compared to normotensive individuals.

## Methods

### Study participants

This was a case-control study nested within participants from the population-based Singapore Chinese Eye Study 2 (SCES-2), the 6-year follow up study from the Singapore Chinese Eye Study 1 (SCES-1). The methodology and recruitment details of the study have been previously reported^19^. In summary, between 2009–2011, SCES-1 recruited 3353 individuals, aged 40–80 years old. Of these, 2661 (87.7%) participated in the SCES-2. A detailed interviewer-administered questionnaire was carried out to collect relevant information about the participants’ medical history along with a physical exam where blood pressure, height and weight were taken.

For the purpose of this study, we selected (1) hypertension cases, defined as participants with hypertension alone and no other significant co-morbidities that could affect retinal vasculature and (2) normal controls with no past medical history of hypertension or other systemic disease and did not meet the criteria for hypertension as outlined below in both the SCES-1 and SCES-2 studies.

Cases were defined to be hypertensive if their systolic blood pressure (BP) was more than or equal to 140 mmHg or diastolic BP more than or equal to 90 mmHg or there was a self-reported history of hypertension or hypertensive medication usage. BP was taken with an automatic blood pressure monitor (Dinamap model Pro Series DP110X-RW, 100V2; GE Medical Systems Information Technologies Inc., Milwaukee, USA). Two readings were taken 5 minutes apart and a third reading was taken if the difference between the first 2 readings was greater than 10 mmHg (systolic) and or 5 mmHg (diastolic)^20^. The age, sex, logMAR visual acuity (VA), intraocular pressure, spherical equivalent, number and type of antihypertensive medications of the patient were also noted. Classes of hypertensive medication in this cohort included angiotensin-converting-enzyme inhibitors, angiotensin II receptor blockers, calcium channel blockers, beta-adrenoreceptor blocking agents, diuretics and alpha-adrenergic agonists. Patients with any systemic illnesses including diabetic mellitus or medications that might affect the retina or had previous cataract surgery, vitrectomies or any obvious retinal pathology that would confound the analysis as determined by trained graders were excluded.

This study adhered to the Declaration of Helsinki. Institutional review board of the Singapore Eye Research Institute approved the ethical aspect of the study and written informed consent was obtained from all eligible participants.

### Imaging protocol

OCTA of the macula was subsequently performed on cases and controls. The AngioVue OCTA system (AngioVue: Optovue, Inc., USA) used in this study is a spectral domain OCTA which uses motion contrast and a split-spectrum amplitude-decorrelation angiography (SSADA) algorithm that allows for the depth-resolved visualisation of the vasculature within the retinal layers and the choroid^21,22^. Built-in software upgrades have improved the signal-to-noise ratio for flow detection and minimized artifacts.

**Figure 1:**
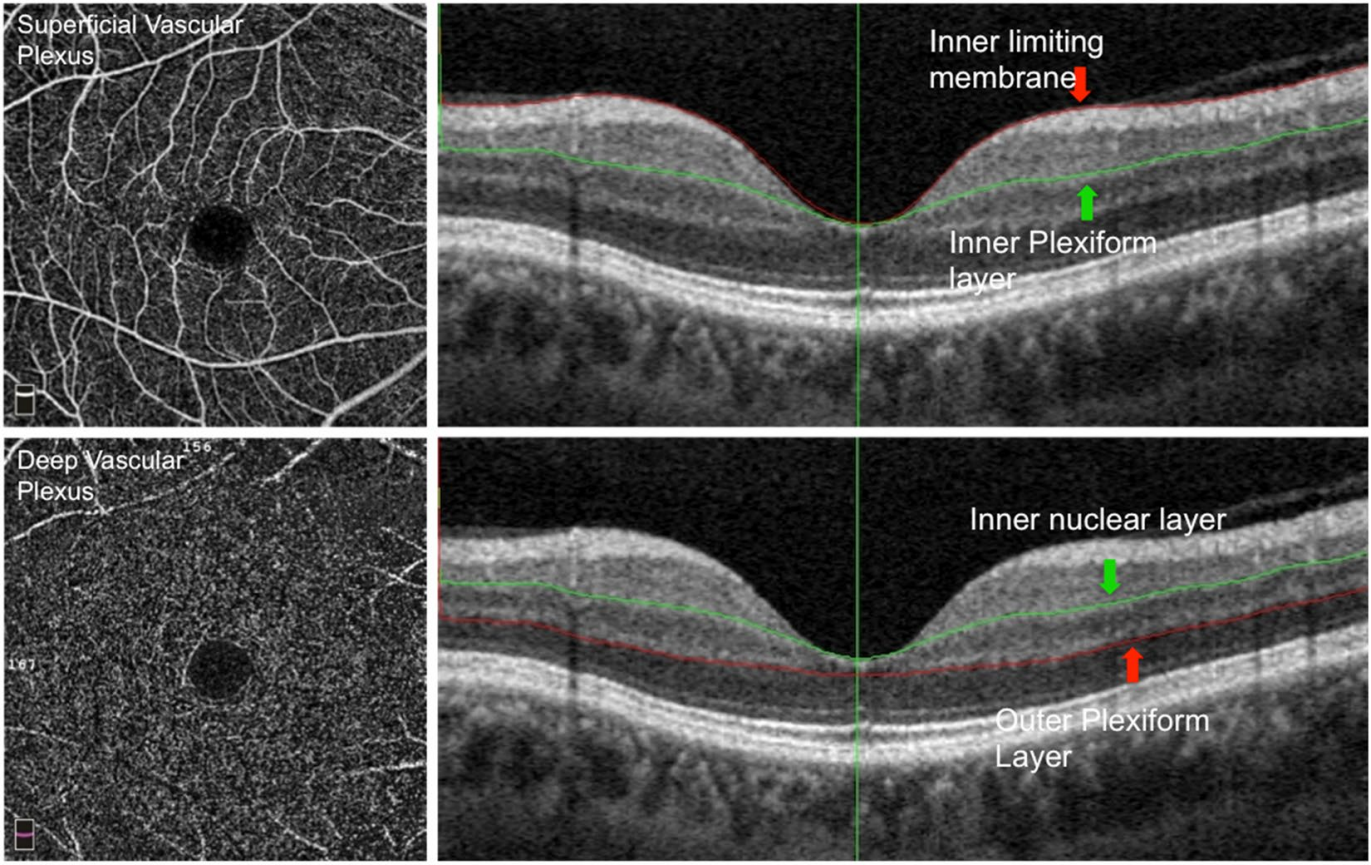
En face OCTA of the superficial (top left) and deep (bottom left) vascular plexus with projection artifact removed with corresponding segmentation lines on the structural OCT showing that the superficial plexus (top right) extends from the inner limiting membrane (red arrow/line) to the inner plexiform layer(green arrow/line) while the deep plexus (bottom left) extends from the inner nuclear layer to the outer plexiform layer.

**Figure 2:**
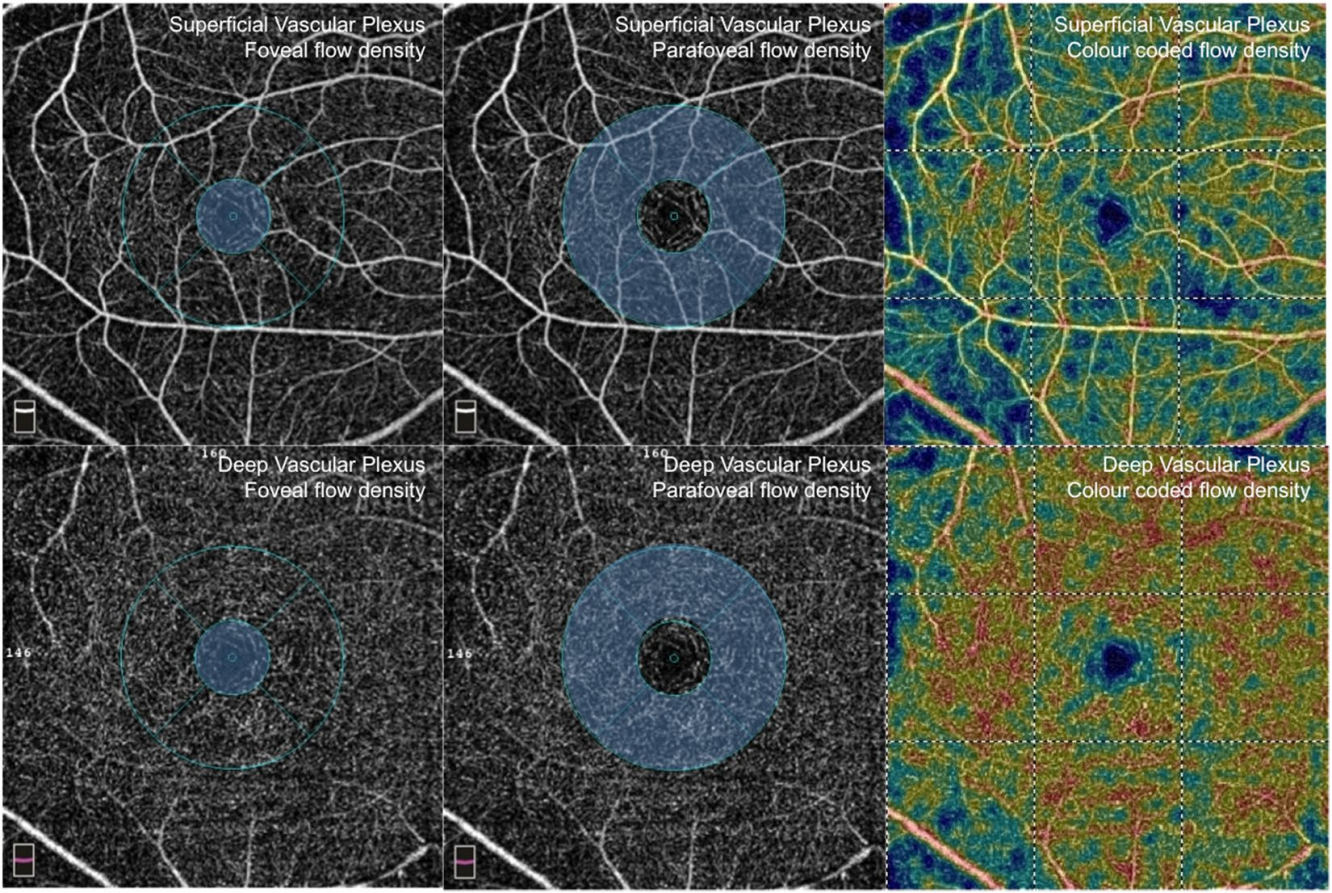
The en face OCT of the superficial (top row) and deep (bottom row) plexus with the areas demarcating the foveal flow density (left) and parafoveal flow density (middle) marked out and the colour coded vessel density map (right).

In this study, 6.0 × 6.0 mm scans centred on the fovea of the right eye were included from each participant. The AngioVue software (version 2016.2.0.35) automatically segmented scans into 4 cross sectional layers but for the purpose of this study only superficial and deep vascular plexuses were studied. Using anatomical landmarks, the level of the SVP was from the inner limiting membrane to the inner plexiform layer and the level of the DVP was from the inner nuclear layer to the outer plexiform layer. The AngioVue AngioAnalytics^TM^ software was used to analyze the FAZ, macula flow, foveal flow density, parafoveal flow density and total flow density at each level. These parameters were derived from an *en face* angiogram of the SVP and DVP (Figs. 1 and 2). Flow quantifies the average flow signal or area of vascularization within region of interest while flow density is percentage of the sample area occupied by vessel lumens following binary reconstruction of images^23,24^. The FAZ was automatically derived using the non-flow area tool of the software. The parafoveal inner retina thickness and parafoveal total retinal thickness were also measured.

Three trained graders who were masked to the participant’s characteristics, ensured the images were of suitable quality to be graded, marked out the vascular area of interest for analysis and ensured that automated segmentation was accurate, manually adjusting it if necessary. As the automated measurement of the deep FAZ was noted to be inaccurate in a large number of scans, manual measurements using ImageJ software were performed for all gradable images by a single grader who was masked to hypertensive status. Anatomically the FAZ is defined as the area within the fovea that is devoid of any retinal vessels. On the OCTA images, this was defined as an area in the fovea region that did not have clear distinct flow signals demarcated by the border between an area of significant flow from retinal vasculature and areas with no flow. Eyes that were found to have any form of retinal pathology that might confound evaluation of the macular architecture including, but not limited to, pigment epithelial detachments, epiretinal membranes, vitreomacular traction and cystoid macula oedema were excluded. In addition, the poor quality images with: (1) very poor signal strength, (2) significant artifact that either obscured the vascular area of interest or obscured more than half the area of the image for analysis and (3) scans with segmentation failure that could not be manually corrected. To establish inter-observer reliability, the 3 graders independently graded the automated superficial and deep macular flow of the same 10 randomly selected subset of participants and a masked second grader repeated 20 (10 control, 10 hypertensive) of the manual deep FAZ measurements. The values collected were analyzed to determine the intraclass correlation coefficient.

### Statistical analysis

Based on a previous study, comparing eyes with chronic hypertension and normal controls, the mean difference of 0.05 mm2 in the superficial FAZ was reported as significant with a maximum standard deviation of 0.7^25^. Hence to detect a significant difference in our study with a power of 80% and a level of significance of 5% (two sided), a minimum of 31 patients in each group (i.e. a total sample size of 62, assuming equal group sizes) is required.

Differences between case and control study patients were tested using independent sample t-tests and Fisher exact tests for continuous and categorical data, respectively. Analysis of covariance (ANCOVA) linear regression models were used to assess the association between hypertensive status and the SVP and DVP vessel flow and flow density, FAZ and parafoveal retinal thickness with odds ratios adjusted for age, sex, intra-ocular pressure, visual acuity, spherical equivalent and mean arterial pressure in multivariate analyses. Linear association between the parafoveal inner retina thickness and other the SVP and DVP vessel flow and density, FAZ were measured using Pearson’s correlation coefficients and partial Pearson’s correlation adjusted for age, sex, intra-ocular pressure, visual acuity, spherical equivalent and mean arterial pressure. Intraclass correlation coefficients (ICC) using a 2 way random effects model were also calculated to determine inter-observer reliability between the 3 graders for the automated measurements and between the 2 graders for the manual deep FAZ measurements. All statistical analyses were performed using R3.3.1 statistical computing language (R Core Team, 2016).

## Results

We recruited 117 cases with hypertension and 64 controls. Of these, 23 hypertension cases and 18 controls were excluded due to poor quality images or retinal pathology. This left 94 (80%) patients in the hypertensive group and 46 (72%) in the control group. For the automated measurements, calculated ICC showed good inter-observer reliability with an ICC of 0.99 [95% CI, 0.98 to 0.99; p < 0.001] and 0.94 [95% CI, 0.61 to 0.99; p < 0.001] for the superficial and deep macular flow respectively. Similarly for the manual deep FAZ measurements, there was good inter-observer reliability with an ICC of 0.92 [95% CI, 0.81 to 0.97; p < 0.001]^26^.

The clinical characteristics of cases and controls are shown in Table 1. There was a significant difference in the mean age of the hypertensive group was 65 ± 9 and 58 ± 5 in the control group [95% CI, P < 0.001]. Females made up 50% and 54% of each group respectively. Participants in the hypertensive group had a mean arterial pressure (MAP) of 98 ± 11 mmHg while the control group had a MAP of 88 ± 7 mmHg which was also statistically significant [95% CI; P < 0.001].

**Table 1:** Comparison of characteristics between Controls and Hypertensives.

Parameter	Controls (n = 46)	Hypertensive (n = 94)	p-value
Age	58.3 (±4.62)	64.77 (±9.03)	**<0.001**
Sex (Female)	25 (54.35%)	47 (50%)	0.72
IOP	14.02 (±2.75)	14.51 (±2.88)	0.334
VA	0.07 (±0.08)	0.11 (±0.09)	**0.009**
Spherical Equivalent	−0.2 (±2.21)	−0.34 (±2.07)	0.727
Mean arterial pressure	88.43 (±6.81)	102.69 (±9.74)	**<0.001**
Systolic blood pressure	123.35 (±11.04)	150.82 (±17.37)	**<0.001**
Diastolic blood pressure	70.97 (±6.62)	78.63 (±8.52)	**<0.001**

In the univariate analysis as shown in Table 2, a number of differences between the hypertensive and control group were noted. The superficial [1.25 ± 0.16 vs 1.13 ± 0.24; 95%CI, P < 0.001] and deep macular flow [1.2 ± 0.18 vs 0.92 ± 0.4; 95%CI, P < 0.001] was reduced in the hypertensive group. Deep parafoveal flow density [59.38 ± 4.46 vs 57.27 ± 5.34; 95%CI, P 0.017] and deep total (foveal + parafoveal) flow density [53.49 ± 5.14 vs 51.05 ± 5.61; 95%CI, P 0.013] were also reduced. Lastly, parafoveal inner retina thickness [121.74 ± 8.3 vs 116.79 ± 9.83; 95%CI, P 0.003] and total parafoveal thickness [318 ± 16.03 vs 311.37 ± 17.82; 95% CI, P 0.03] were also reduced in the hypertensive group.

**Table 2:** Univariate comparison of retinal parameters between Controls and Hypertensives.

Parameter	All participants (n = 140)	Controls (n = 46)	Hypertensive (n = 94)	p-value
**Superficial**				
Foveal avascular zone	0.4 (±0.15)	0.39 (±0.12)	0.4 (±0.17)	0.529
Macula flow	1.17 (±0.23)	1.25 (±0.16)	1.13 (±0.24)	**<0.001**
Foveal flow density	28.93 (±5.91)	28.78 (±5.81)	29.01 (±5.99)	0.832
Parafoveal flow density	47.8 (±4.79)	48.89 (±4.65)	47.26 (±4.79)	0.06
Total flow density	45.17 (±3.48)	45.85 (±3.21)	44.84 (±3.57)	0.101
**Deep**				
Manual foveal avascular zone	0.34 (±0.1)	0.32 (±0.11)	0.35 (±0.1)	0.172
Manual foveal avascular zone perimeter	2.22 (±0.35)	2.14 (±0.38)	2.27 (±0.33)	0.087
Macula flow	1.01 (±0.37)	1.2 (±0.18)	0.92 (±0.4)	**<0.001**
Foveal flow density	27.81 (±6.75)	27.94 (±6.35)	27.75 (±6.97)	0.873
Parafoveal flow density	57.97 (±5.14)	59.38 (±4.46)	57.27 (±5.34)	**0.017**
Total flow density	51.86 (±5.56)	53.49 (±5.14)	51.05 (±5.61)	**0.013**
Parafoveal inner retina thickness	118.45 (±9.61)	121.74 (±8.3)	116.79 (±9.83)	**0.003**
Total parafoveal thickness	313.6 (±17.46)	318 (±16.03)	311.37 (±17.82)	**0.03**

**Figure 3:**
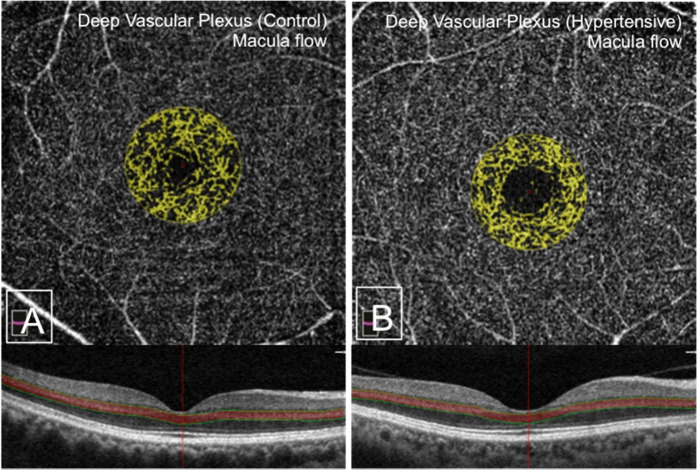
OCT-A images showing the difference in the deep macula flow between the control group (**A**) and hypertensive group (**B**).

**Figure 4:**
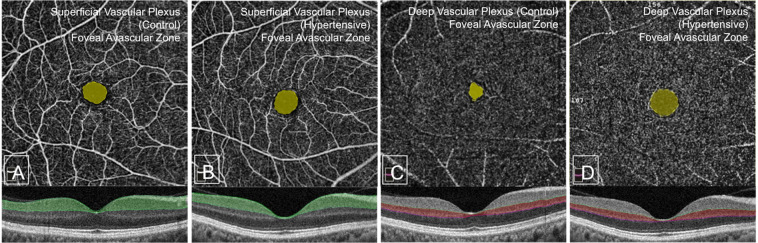
The 2 images on the left show that there is no significant difference in the size of the superficial FAZ between the control (**A**) and hypertensive (**B**) groups respectively. The 2 images on the right show the difference in the size of the deep FAZ between the control (**C**) and hypertensive groups (**D**), with the FAZ of the control group being significantly smaller.

Previous studies have shown changes in the vessel density and FAZ in the SVP and/or DVP with age and sex as well as changes in image magnification with differing axial length^27–30^. Thus we adjusted for age, sex, intraocular pressure (IOP), logMAR visual acuity, spherical equivalent and MAP. The multivariate analysis (Table 3) showed that only the decrease in the macular flow in the deep layer [1.21 ± 0.1 vs 0.92 ± 0.2; 95%CI, P 0.002] seen in the univariate analysis was still significant (Fig. 3). However, an additional significant difference was noted in that of an increase in the size of the deep FAZ [3.15 ± 0.3 vs 3.47 ± 0.38; 95%CI, P 0.03] and deep FAZ perimeter [21.4 ± 1.1 vs 22.74 ± 1.35; 95%CI, P 0.016] in the hypertensive group (Fig. 4). No significant difference was noted in the superficial FAZ. Differences in the deep parafoveal and total (foveal + parafoveal) density, parafoveal inner retina and parafoveal total retina thickness were no longer significant.

**Table 3:** Multivariate ^¶^ comparison of retinal parameters between Controls and Hypertensives.

Parameter	Controls (n = 46)	Hypertensive (n = 94)	p-value
**Superficial**			
Foveal avascular zone	0.38 (±0.05)	0.4 (±0.07)	0.059
Macula flow	1.25 (±0.04)	1.13 (±0.09)	0.084
Foveal flow density	29.03 (±1.15)	29.04 (±1.59)	0.19
Parafoveal flow density	48.81 (±0.74)	47.15 (±0.88)	0.359
Total flow density	45.82 (±0.98)	44.78 (±1.31)	0.426
**Deep**			
Manual foveal avascular zone	3.15 (±0.3)	3.47 (±0.38)	0.030
Manual foveal avascular zone perimeter	21.4 (±1.1)	22.74 (±1.35)	0.016
Macula flow	1.21 (±0.1)	0.92 (±0.2)	0.002
Foveal flow density	28.18 (±1.74)	27.84 (±1.87)	0.674
Parafoveal flow density	59.42 (±1.29)	57.25 (±1.73)	0.327
Total flow density	53.54 (±2.13)	51.08 (±2.79)	0.178
Parafoveal inner retina thickness	121.84 (±3.47)	116.81 (±5.12)	0.572
Total parafoveal thickness	318.86 (±6.18)	311.65 (±7.86)	0.952

^¶^Adjusted for age, sex, IOP (right eye), log Mar visual acuity, spherical equivalent, mean arterial pressure.

The breakdown of the medications classes can be seen in Supplementary Table 1. No significant correlation between visual acuity and any of the OCTA measured adjusted parameters were reported (Supplementary Table 2). A significant correlation between the mean arterial pressure reading and the adjusted foveal flow density was noted however the other adjusted flow and flow density parameters were not different (Supplementary Table 3).

## Discussion

In this population-based case control study, we found that deep macular flow is decreased and the size and perimeter of the deep FAZ are increased in participants with hypertension compared to normal controls. Recent interests in the assessment of micro and macro vascular damage in hypertensive patients show that capillary rarefaction may be related to end organ damage and increased cardiovascular risk^31–33^.

In healthy normotensive subjects, previous studies show that subclinical microvascular changes and vascular rarefaction in the kidneys and the skin are seen in patients who have a higher risk of developing hypertension and subsequent cardiovascular disease^4,34–38^. Previous studies using colour fundus photos showed that narrower arterioles, wider venules and features such as arterio-venous nicking, vessel tortuosity and fractal dimensions were associated with increased risk of hypertension, cardiovascular and cerebrovascular disease, cognitive decline and even chronic kidney disease in some populations^10,39–42^. Studies using laser Doppler flowmetry showed retinal capillary rarefaction was present in eyes with hypertension, with a greater intercapillary distance, reduced capillary area as well as reduced retinal capillary flow in untreated hypertensive patients and these changes increased with the duration of disease^43,44^. Hence, using non-invasive retinal vascular imaging to quantify these vascular changes, has the potential to identify subclinical and early hypertensive changes, risk-stratify normotensive patients and allow for early treatment to prevent end organ damage and cardiovascular complications. Compared with the aforementioned imaging modalities, OCTA has potential advantages such as better resolution, with the ability to image smaller caliber vessels including capillaries, as well as depth resolution allowing assessment of the both the superficial, intermediate and deep vascular plexus^15,17^.

In our study, eyes with hypertension were found on OCTA to have significant alterations in the DVP but not the SVP. The preferential loss of the DVP has been seen in several other pathologies including diabetic retinopathy as well as in retinal vein occlusions^45^. In a study by *Dupas et al*. they found that the vessel density in the DVP was more affected compared to the superficial plexus in patients with diabetic retinopathy and poorly controlled type 1 diabetes. Several other studies have made similar findings in both type 1 and 2 diabetes, some of which also purport that it is the ischemia at the level of the DVP that results in outer retina and photoreceptor disruption^46–49^.

The mechanism that results in this preferential loss is still undetermined but theories have suggested that the DVP is the conduit by which blood from the SVP drains into the deep venules and where more retinal capillary units terminate, making it more distal from the arterial circulation and hence more susceptible to disruption in retinal blood flow^46,50,51^. However, it must be noted that a number of other studies looking at pre-clinical diabetic retinopathy to proliferative diabetic retinopathy did not find similar a predilection for the DVP and found that both the SVP and DVP were similarly affected^52,53^. In the context of retinal vein occlusions, a number of studies have shown that the DVP is more severely affected in comparison to the SVP. A possible theory postulated that this may be because the rise hydrostatic pressure is faster and more severe in the DVP resulting in a perfusion decrease^45,54,55^. The study of DVP versus SVP alterations may have important clinical implications as it may have a greater effect on the outer retinal layers and this may affect visual functioning, however more studies are needed to verify this relationship. It must be considered also that in all studies examining quantitative changes in the SVP and DVP separately, accurate segmentation and removal of projection artifact is essential and this can sometimes be challenging in eyes with significant diseases that alter the anatomical retinal layers.

Outer retinal losses have been reported previously shown to be associated a with an increase FAZ^47^. Although in our study increase in both the FAZ area and perimeter was noted, no relationship was found between VA and the FAZ or any other measured vasculature parameter (Supplementary Table B). However, of note in our study, outer retinal changes, which may show better correlation to visual function were not specifically graded. More studies need to be done to establish the effect of retinal microvascular changes seen in hypertensive eyes and its impact on the outer retina, photoreceptors and ultimately visual function. Similar to a previous study by Kong *et al*. on OCT findings in hypertensive eyes, a decrease in peri-foveal macular thickness was observed in the univariate analysis however this was no longer significant in the between the hypertensive and control groups in our multivariate analysis^56^. In contrast to another cross-sectional cohort study by Lee *et al*. which found a reduction in the foveal vessel density, perfusion density, mean GC-IPL thickness, CFT and RNFL and an increase in the FAZ at the level of the SVP in the hypertensive group compared to controls, our paper did not find any significant differences between the 2 groups at the level of the SVP. However, their paper may not be comparable to ours because no data on the DVP was presented in their paper and the OCTA macular scan area used was 3 × 3 mm on the Zeiss Angioplex System compared to 6 × 6 mm scans Optovue Angiovue scans used in our study^25^. Also, the definition of hypertension differed in their study with their 2 comparative groups having either (1) chronic hypertension >10 years and (2) relieved grade IV hypertensive retinopathy for at least 1 year. They also did not adjust their analysis for variables such as age, sex and refractive error and also did not take into account anti-hypertensive medication treatment. Our findings were similar to the study done by Chua *et al*., in that both our univariate analysis’ found a decrease in the deep capillary density. However, the difference is that in our study after adjusting for IOP, visual acuity, spherical equivalent and MAP in the multivariate analysis, there was no longer a significant difference in this parameter. In addition, our study also looked at the macula flow and FAZ in the SVP and DVP which was not analyzed in the study by Chua *et al*.

Hence, OCTA allows for a non-invasive, efficient and increasingly widely available method of imaging method the retinal microvasculature with advantages compared to conventional injectable dye angiography, which is unsuitable for use in large cohort studies and for consecutive follow up visits^17^. Apart from blood pressure reduction, targeting the microcirculation itself could be beneficial in preventing or reducing end organ damage and thus reducing morbidity and mortality^4^. Other current methods of microvasculature imaging available for other organs such as nailbed capiloroscopy, contrast based cardiovascular angiography or cerebrovascular angiography have their limitations and are associated with higher risks. So far no other widely available imaging modality has been able to measure microvasculature with such ease, reproducibility and resolution compared to OCTA of the retina^57^. Hence, studying changes in the microvasculature on OCTA in relation to systemic vascular disease may have the potential to risk stratify, detect early disease, monitor disease progression and detect improvements in vasculature rarefaction in response to treatment.

Our study strengths are that this study, to our knowledge, is the largest cohort of both hypertensive and control eyes with standardized imaging protocols and automated quantitative analysis of retinal microvascular parameters which have previously proven to have good reproducibility as well as good inter-observer agreement between graders^17,58,59^. However, there are a few limitations to this study. Firstly, hypertension was defined as a patient reported history of hypertension, the use of anti-hypertensives or a single setting reading of increased BP, ambulatory blood pressure was not measured in this study and hence a small number of patients who may have hypertension could have been missed and blood pressure control was not assessed. Secondly, our changes in retinal vasculature was not correlated with other peripheral microvasculature parameters, renal function, body mass index or other end organ damage measurements. Our current OCTA imaging depends on motion contrast from blood flow to visualize the retinal vasculature, hence vessels with flow below the detectable threshold may not be able to be imaged^60^. We acknowledge that our delimited FAZ may contain some background speckles of flow but this was assumed to be insignificant background noise artifact. In addition, the image artifacts seen on OCTA that obstruct an accurate analysis of the microvasculature had to be excluded, however these only involved a small proportion of the overall cohort and is unlikely to significantly affect our results. Also, our cohort only included participants who were ethnically Chinese and these results may not be applicable to other races.

While we noted the number and classes of hypertensive agents, we did not do any subgroup analysis correlating type and number of medications with OCTA parameters due to insufficient numbers. This is a possible area for future research as improvement in vascular rarefaction has been reported with the use of various anti-hypertensive agents and it would be interesting to note if there were differences in improvement in rarefaction between classes of anti-hypertensives^4,35,44^.

In conclusion, we have shown that there is an association between systemic hypertension and decreased retinal flow and increased FAZ area and perimeter in the DVP seen on OCTA. However, further studies are needed verify these findings and to study its potential clinical implications as a clinical tool for monitoring hypertensive end organ damage and for risk stratification of normotensive patients.

## Supplementary information

Supplementary Information.

Supplementary Information 2.

Supplementary Information 3.
