# Supplementary material for: Systemic hypertension associated retinal microvascular changes can be detected with optical coherence tomography angiography
Source: Sci Rep. 2020 Jun 12;10:9580. doi: 10.1038/s41598-020-66736-w (PMC7293289; doi:10.1038/s41598-020-66736-w)
Supplement: Supplementary file 1 — Supplementary Information. [file 41598_2020_66736_MOESM1_ESM.docx]

**Full title:** Systemic hypertension associated retinal microvascular changes can be detected with optical coherence tomography angiography

**Authors:**

Christopher Sun^1^, Carlo Ladores^1,2^, Jimmy Hong^3^, Nguyen Duc Quang^3^, Jacqueline Chua^1,3^, Daniel Ting^1^, Leopold Schmetterer^2^, Wong Tien Yin^1^, Cheng Ching Yu^2^, Anna C.S. Tan^1^

^1^ Singapore National Eye Centre

^2^ University of Santo Tomas Hospital, Manila, Philippines

^3^Singapore Eye Research Institute

***Supplementary Table 1. Analysis of number and class of anti-hypertensive medications in the hypertensive cohort***

| Medication | | Number | Percentage |
| --- | --- | --- | --- |
| 1 anti-hypertensive (n = 31) | |  |  |
|  | Calcium channel blocker | 12 | 39% |
|  | Angiotensin II receptor blocker | 9 | 29% |
|  | Beta-receptor blocker | 5 | 16% |
|  | Angiotensin converting enzyme inhibitors | 3 | 10% |
|  | Alpha Receptor Antagonist | 1 | 3% |
|  | Diuretics | 1 | 3% |
| ≥2 anti-hypertensives (n = 11) | |  |  |
|  | Beta receptor blockers + Calcium channel blockers | 3 | 27% |
|  | Beta receptor blockers + Angiotensin converting enzyme inhibitors | 2 | 18% |
|  | Beta receptor blockers + Angiotensin II receptor blockers | 1 | 9% |
|  | Beta receptor blockers + Unspecified anti-hypertensive medication | 1 | 9% |
|  | Alpha receptor antagonist + Angiotensin II receptor blocker | 1 | 9% |
|  | Alpha receptor antagonist +Calcium channel blocker | 1 | 9% |
|  | Calcium channel blockers + Angiotensin II receptor blocker | 1 | 9% |
|  | Beta receptor blocker + calcium channel blocker + angiotensin II receptor blocker | 1 | 9% |
