# Supplementary material for: Systemic hypertension associated retinal microvascular changes can be detected with optical coherence tomography angiography
Source: Sci Rep. 2020 Jun 12;10:9580. doi: 10.1038/s41598-020-66736-w (PMC7293289; doi:10.1038/s41598-020-66736-w)
Supplement: Supplementary file 2 — Supplementary Information 2. [file 41598_2020_66736_MOESM2_ESM.docx]

**Full title:** Systemic hypertension associated retinal microvascular changes can be detected with optical coherence tomography angiography

**Authors:**

Christopher Sun^1^, Carlo Ladores^1,2^, Jimmy Hong^3^, Nguyen Duc Quang^3^, Jacqueline Chua^1,3^, Daniel Ting^1^, Leopold Schmetterer^2^, Wong Tien Yin^1^, Cheng Ching Yu^2^, Anna C.S. Tan^1^

^1^ Singapore National Eye Centre

^2^ University of Santo Tomas Hospital, Manila, Philippines

^3^Singapore Eye Research Institute

***Supplementary Table 2. Correlation coefficients between Visual Acuity (logMar) and OCTA parameters***

| Parameter | | | Coefficient (95% CI) | | p-value |
| --- | --- | --- | --- | --- | --- |
| Superficial | | |  | |  |
|  | Foveal avascular zone | |  | |  |
|  |  | Unadjusted | 0.16 | | 0.059 |
|  |  | Adjusted**^¶^** | 0.074 | | 0.392 |
|  | Macula flow | |  | |  |
|  |  | Unadjusted | -0.194 | | **0.022** |
|  |  | Adjusted**^¶^** | -0.019 | | 0.828 |
|  | Foveal flow density | |  | |  |
|  |  | Unadjusted | -0.062 | | 0.469 |
|  |  | Adjusted**^¶^** | -0.079 | | 0.363 |
|  | Parafoveal flow density | |  | |  |
|  |  | Unadjusted | -0.07 | | 0.413 |
|  |  | Adjusted**^¶^** | -0.014 | | 0.868 |
|  | Total flow density | |  | |  |
|  |  | Unadjusted | -0.056 | | 0.511 |
|  |  | Adjusted**^¶^** | 0.042 | | 0.632 |
| Deep | | |  | |  |
|  | Manual foveal avascular zone | |  | |  |
|  |  | Unadjusted | 0.135 | | 0.113 |
|  |  | Adjusted**^¶^** | 0.146 | | 0.091 |
|  | Manual foveal avascular zone perimeter | | |  |  |
|  |  | Unadjusted | 0.138 | | 0.105 |
|  |  | Adjusted**^¶^** | 0.146 | | 0.092 |
|  | Macula flow | |  | |  |
|  |  | Unadjusted | -0.24 | | **0.004** |
|  |  | Adjusted**^¶^** | -0.057 | | 0.513 |
|  | Foveal flow density | |  | |  |
|  |  | Unadjusted | -0.145 | | 0.087 |
|  |  | Adjusted**^¶^** | -0.116 | | 0.18 |
|  | Parafoveal flow density | |  | |  |
|  |  | Unadjusted | -0.204 | | **0.016** |
|  |  | Adjusted**^¶^** | -0.08 | | 0.356 |
|  | Total flow density | |  | |  |
|  |  | Unadjusted | -0.233 | | **0.006** |
|  |  | Adjusted**^¶^** | -0.084 | | 0.33 |
| Total parafoveal thickness | | |  | |  |
|  | | Unadjusted | -0.074 | | 0.383 |
|  | | Adjusted**^¶^** | 0.077 | | 0.378 |

**^¶^**adjusted for age, sex, IOP, logMAR visual acuity, spherical equivalent and mean arterial pressure
