# Supplementary material for: Systemic hypertension associated retinal microvascular changes can be detected with optical coherence tomography angiography
Source: Sci Rep. 2020 Jun 12;10:9580. doi: 10.1038/s41598-020-66736-w (PMC7293289; doi:10.1038/s41598-020-66736-w)
Supplement: Supplementary file 3 — Supplementary Information 3. [file 41598_2020_66736_MOESM3_ESM.docx]

**Full title:** Systemic hypertension associated retinal microvascular changes can be detected with optical coherence tomography angiography

**Authors:**

Christopher Sun^1^, Carlo Ladores^1,2^, Jimmy Hong^3^, Nguyen Duc Quang^3^, Jacqueline Chua^1,3^, Daniel Ting^1^, Leopold Schmetterer^2^, Wong Tien Yin^1^, Cheng Ching Yu^2^, Anna C.S. Tan^1^

^1^ Singapore National Eye Centre

^2^ University of Santo Tomas Hospital, Manila, Philippines

^3^Singapore Eye Research Institute

***Supplementary Table 3. Correlation coefficients between Mean arterial pressure and OCTA parameters***

| Parameter | | | Coefficient (95% CI) | p-value |
| --- | --- | --- | --- | --- |
| Superficial | | |  |  |
|  | FAZ | |  |  |
|  |  | Unadjusted | -0.1 | 0.24 |
|  |  | Adjusted**^¶^** | -0.129 | 0.135 |
|  | Mac flow | |  |  |
|  |  | Unadjusted | -0.051 | 0.55 |
|  |  | Adjusted**^¶^** | -0.003 | 0.971 |
|  | Foveal flow density | |  |  |
|  |  | Unadjusted | 0.182 | **0.031** |
|  |  | Adjusted**^¶^** | 0.185 | **0.031** |
|  | Parafoveal flow density | |  |  |
|  |  | Unadjusted | -0.096 | 0.259 |
|  |  | Adjusted**^¶^** | -0.078 | 0.367 |
|  | Total flow density | |  |  |
|  |  | Unadjusted | -0.03 | 0.729 |
|  |  | Adjusted**^¶^** | 0.027 | 0.755 |
| Deep | | |  |  |
|  | Manual FAZ | |  |  |
|  |  | Unadjusted | 0.022 | 0.796 |
|  |  | Adjusted**^¶^** | 0.029 | 0.734 |
|  | Manual FAZ perimeter | |  |  |
|  |  | Unadjusted | 0.016 | 0.85 |
|  |  | Adjusted**^¶^** | 0.027 | 0.752 |
|  | Mac flow | |  |  |
|  |  | Unadjusted | -0.024 | 0.78 |
|  |  | Adjusted**^¶^** | 0.028 | 0.747 |
|  | Foveal flow density | |  |  |
|  |  | Unadjusted | 0.097 | 0.255 |
|  |  | Adjusted**^¶^** | 0.09 | 0.297 |
|  | Parafoveal flow density | |  |  |
|  |  | Unadjusted | -0.075 | 0.382 |
|  |  | Adjusted**^¶^** | -0.036 | 0.68 |
|  | Total flow density | |  |  |
|  |  | Unadjusted | -0.009 | 0.914 |
|  |  | Adjusted**^¶^** | 0.056 | 0.519 |
| Total parafoveal thickness | | |  |  |
|  | | Unadjusted | -0.203 | 0.016 |
|  | | Adjusted**^¶^** | -0.205 | 0.017 |

**^¶^**adjusted for age, sex, IOP, logMAR visual acuity, spherical equivalent
